# A survey of training and practice patterns of massage therapists in two US states

**DOI:** 10.1186/1472-6882-5-13

**Published:** 2005-06-14

**Authors:** Karen J Sherman, Daniel C Cherkin, Janet Kahn, Janet Erro, Andrea Hrbek, Richard A Deyo, David M Eisenberg

**Affiliations:** 1 Center for Health Studies, Group Health Cooperative, Seattle, Washington 98101, USA; 2 Department of Epidemiology, University of Washington, Seattle, Washington 98195, USA; 3 Departments of Family Medicine and Health Services, University of Washington, Seattle, Washington 98195, USA; 4 Department of Psychiatry, University of Vermont, Burlington, Vermont, 05405, USA; 5 Harvard Medical School Osher Institute and Division for Research and Education in Complementary and Integrative Medical Therapies, Harvard Medical School, Boston, Massachusetts 02215, USA; 6 Departments of Medicine and Health Services, University of Washington, Seattle, Washington, USA

## Abstract

**Background:**

Despite the growing popularity of therapeutic massage in the US, little is known about the training or practice characteristics of massage therapists. The objective of this study was to describe these characteristics.

**Methods:**

As part of a study of random samples of complementary and alternative medicine (CAM) practitioners, we interviewed 226 massage therapists licensed in Connecticut and Washington state by telephone in 1998 and 1999 (85% of those contacted) and then asked a sample of them to record information on 20 consecutive visits to their practices (total of 2005 consecutive visits).

**Results:**

Most massage therapists were women (85%), white (95%), and had completed some continuing education training (79% in Connecticut and 52% in Washington). They treated a limited number of conditions, most commonly musculoskeletal (59% and 63%) (especially back, neck, and shoulder problems), wellness care (20% and 19%), and psychological complaints (9% and 6%) (especially anxiety and depression). Practitioners commonly used one or more assessment techniques (67% and 74%) and gave a massage emphasizing Swedish (81% and 77%), deep tissue (63% and 65%), and trigger/pressure point techniques (52% and 46%). Self-care recommendations, including increasing water intake, body awareness, and specific forms of movement, were made as part of more than 80% of visits. Although most patients self-referred to massage, more than one-quarter were receiving concomitant care for the same problem from a physician. Massage therapists rarely communicated with these physicians.

**Conclusion:**

This study provides new information about licensed massage therapists that should be useful to physicians and other healthcare providers interested in learning about massage therapy in order to advise their patients about this popular CAM therapy.

## Background

Although massage is one of the oldest healthcare practices in the world, with references to it found in ancient Chinese medical texts as well as in the writings of Hippocrates, medical doctors in the US have not practiced therapeutic massage for nearly 100 years [1]. In the 1930's and 1940's, massage fell out of favor with nurses and physical therapists as well. However, since the 1970's, interest in massage therapy has burgeoned and it is now one of the most popular complementary and alternative medical (CAM) modalities. In the US, Eisenberg, et al. [2] found 11% of randomly surveyed Americans had used massage for treating common medical conditions in 1997, with 62% of these receiving massage from a trained massage therapist. They found that the number of visits to massage therapists exceeded that to all other CAM providers except chiropractors, with trained massage therapists providing an estimated 114 million massage sessions to Americans in 1997. Eighty percent of randomly surveyed US adults with health insurance claimed they would be "likely" to use massage, making it the most popular of the 11 therapies included in the survey [3]. Palinkas [4] reported that massage was the third most commonly used type of CAM among primary care patients, with 17.2% of CAM users reporting use of massage within the last year for the same reason they were seeking primary care.

Despite this growth in the popularity of massage, little is known about the practices of licensed massage therapists. We included massage therapists in our study of random samples of licensed CAM practitioners and their practices [5,6]. In this report, we present new information about the demographic and training characteristics of licensed massage therapists, the reasons patients seek their care, the assessment process massage therapists use during visits, and the treatments and self-care recommendations they provide. We have included information about massage efficacy and safety and communication between massage therapists and physicians in the Discussion section to assist biomedical healthcare providers in placing our findings in the broader context of patient care.

## Methods

### Original study

The data presented in this paper were collected as part of a larger study of four licensed CAM professions, including massage therapy. The methods are described in detail elsewhere [5,6] and summarized here. Our goal was to obtain data on 20 consecutive visits to 50 randomly selected massage therapists in one Northeastern state (Connecticut) and one Western state (Washington) who gave at least 10 massage treatments per week. Massage therapists were randomly sampled from state licensure listings in Washington (1998) and Connecticut (1999). In both states, licensing requirements for massage therapists including having 500 hours of education and a passing score on the national examination. We excluded providers without identifiable telephone numbers and those not currently practicing. The proportion of ineligible practitioners was 47% in Connecticut and 33% in Washington. About 84% of ineligible Connecticut massage therapists lacked identifiable phone numbers, while in Washington ineligible therapists were about equally divided between those who were not practicing and those who lacked identifiable phone numbers.

All participating massage therapists were interviewed about their demographic, training, and practice characteristics. Those with at least 10 visits in a typical week were then invited to participate in visit-based data collection. A sample of those seeing 5 to 9 visits per week were also invited to collect data on patient visits. Massage therapists with less than 5 visits per week were not asked to collect visit data and provided about 2% of all massage visits [6].

We obtained approval from the Group Health Cooperative, University of Washington, and Beth Israel Deaconess Medical Center Institutional Review Boards. Visit data were collected between May and September in 1998 in Washington and between June 1999 and March 2000 in Connecticut. Massage therapists were given visit forms marked with unique identification codes and were asked to record data on 20 consecutive visits (even if the same patient was seen more than once). Practitioners were randomly assigned weekdays to begin data collection.

### Visit form

The one-page visit form was modeled after those used in the National Ambulatory Medical Care Survey (NAMCS) [7] and a copy of the visit form is found in Additional File 1. Whenever possible, questions were worded identically to those in the NAMCS (e.g., demographic characteristics, smoking status, reason for visit, referral source, source of payment, visit duration, visit disposition). New questions asked if the patient was receiving care from a conventional medical provider for the primary problem and if the massage therapist had communicated about this problem with a conventional provider who also provided care for the patient's main problem. We also designed special questions to capture information about massage treatments, including information on use of specific assessment techniques, massage techniques, and lifestyle recommendations. We asked practitioners to record up to five "complaints, symptoms, or other reasons for this visit" using the patients own words, listing the most important complaint or reason first. These data were classified using the NAMCS Reason for Visit Classification System, which distinguishes among symptoms, diseases, diagnostic/screening/preventive interventions, treatments, and injuries [7]. Individual reasons for visit were then clustered into larger categories that correspond to *International Classification of Diseases, Ninth Edition *(ICD9) chapters. No information was collected on adverse experiences as part of this study.

### Analysis

In the massage therapist analyses, Chi-square and Fisher Exact tests were used to compare proportions, and Kruskal Wallis tests were used to compare medians. Even though standard errors are not presented, they are always within 5 percentage points of the estimate. Analyses were performed using SAS version 8 (SAS Institute, Cary, NC).

In the visit analyses, each visit in the sample was weighted by the inverse of its sampling probability, which reflected both the chance that the particular provider participated and the estimated proportion of that provider's annual visits included in the study. Consequently, our results represent estimates of all visits made to massage therapists in each state, except for the 2% of visits made to providers with fewer than 5 visits per week or visits to therapists who were not licensed. Because of the two-stage sampling design, we used SUDAAN software (Research Triangle Institute, Research Triangle, NC) to calculate standard errors and confidence intervals using Taylor series linearization. Because of the large sample sizes (965 and 1040 visits) the weighted percentages presented in the tables have small standard errors, generally between 0.5 and 2.5 percentage points and rarely exceeding 3 percentage points. As a result, moderate to large differences between the states are also statistically significant. Therefore, the standard errors are not included in the tables.

## Results

### Participation rates

Participation rates for the massage therapist interview were 86% (114 of 133) in Connecticut and 84% in Washington (112 of 134). Of the massage therapists who saw enough clients per week to be eligible to collect visit data, 66% in Connecticut (61 of 93) and 70% in Washington (65 of 93) complied. Data were collected on 965 visits in Connecticut and 1040 visits in Washington.

### Characteristics of the massage therapists

In both states, massage therapists were typically white, female and had a median age of 42 years (Table 1). Virtually all of them received their basic training in the US, with most having trained in the state where they were currently practicing. A small fraction had no formal training. In both states, massage therapists reported training a median of about 600 hours. Massage therapists reported a median of 4 to 5 years in practice, with only 18% in Connecticut and 13% in Washington reporting more than 10 years.

**Table 1:** Demographic and training characteristics of massage therapists

	State		
	
	Connecticut	Washington	
	
	(N = 114 practitioners)	(N = 112 practitioners)	p value
*Demographic Characteristics*			
Women	85%	85%	
White	95%	95%	
Hispanic	4%	4%	
Median Age	41.5 yrs.	41.5 yrs.	
*Basic Training*			
Formal Schooling	93%	94%	
US – other states	12%	8%	
US – same state	81%	85%	
Foreign	1%	1%	
No Formal Schooling	6%	6%	
*Median Years in Practice*	5 yrs.	4 yrs.	
*Post-graduate Training*			
Any	79%	52%	***
Craniosacral	14%	12%	
Neuromuscular	10%	10%	
Reflexology	10%	6%	
Reiki	13%	6%	
Polarity	5%	5%	
Lymph Drainage	3%	5%	
Meridian – based (Shiatsu, Tuina, acupressure)	22%	10%	*
Myofascial Release	14%	3%	**
Pregnancy Massage	6%	1%	

* p < 0.05; ** p < 0.01; *** p < 0.001

Most massage therapists (82% in Connecticut and 89% in Washington) reported additional hours of training after graduation, receiving a median of 60 hours. Nearly 80% of the massage therapists in Connecticut and about half in Washington reported "specialty or advanced training" (i.e., continuing education), with 43% and 31%, respectively, reporting multiple types of such training. Continuing education was extremely heterogeneous, with practitioners noting 56 different types of training in Connecticut and 37 types in Washington. However, only 4 types of training were received by more than 10% of practitioners in Connecticut (meridian -based therapies, craniosacral, myofascial release and Reiki) and only one type of training was received by more than 10% of practitioners in Washington (craniosacral therapy) (Table 1). Ten percent of massage therapists in Connecticut and 8% of those in Washington held other healthcare profession licenses. All but one of those (acupuncture) were in biomedical areas, most commonly nursing.

Connecticut massage therapists reported a median of 10 patient visits per week and 12 hours of direct patient care per week, compared with 15 patient visits per week and 17 hours of direct patient care, for massage therapists in Washington (p < 0.02 for hours of direct patient care).

### Reasons for visits to massage therapists

Visits to massage therapists were for a limited number of conditions. About 60% of visits were for musculoskeletal symptoms, particularly back, neck, and shoulder symptoms (Table 2). Visits for "wellness" (i.e., relaxation) accounted for another 20% of visits and mental health concerns, largely anxiety and depression, for another 6 to 9% of visits. Virtually all other visits were for general body symptoms (mostly generalized pain) or "nervous system" symptoms (most commonly headache).

**Table 2:** Most common reasons for visits to massage therapists licensed in Connecticut (1999) and Washington (1998) by broad and specific categorization

Connecticut			Washington		
(N = 965 visits)			(N = 1040 visits)		

Broad Categories*	% with Primary Reason		Broad Categories*	% with Primary Reason	
			
1. Musculoskeletal Symptoms	59.2		1. Musculoskeletal Symptoms	63.0	
2. Wellness**	19.5		2. Wellness**	18.7	
3. Psychological and Mental Health Symtoms	8.8		3. Psychological and Mental Health Symtoms	5.7	
4. General Symptoms	4.5		4. Nervous System Symptoms	4.9	
5. Nervous System Symptoms	3.7		5. General Symptoms	3.7	
Top 5 Categories	95.7		Top 5 Categories	96.0	
	% with			% with	
Specific Reasons	Primary Reason	Any Reason	Specific Reasons	Primary Reason	Any Reason
1. Back Symptoms	20.4	34.4	1. Back Symptoms	20.2	39.8
2. Massage Wellness	19.5	25.8	2. Neck Symptoms	20.0	38.5
3. Neck Symptoms	13.0	24.1	3. Massage Wellness	18.7	26.5
4. Shoulder Symptoms	8.4	23.1	4. Shoulder Symptoms	7.4	26.6
5. Anxiety or Depression	8.8	17.4	5. Anxiety or Depression	5.2	12.3
6. Leg Symptoms	5.0	10.0	6. Headache	3.7	8.4
7. Unspecified Muscle Symptoms	4.0	6.3	7. Leg Symptoms	2.6	6.3
8. Generalized Pain	3.1	4.5	8. Generalized Pain	2.1	3.5
9. Headache	1.6	5.2	9. Hip Symptoms	1.9	6.7
10. Unspecified Joint Symptoms	1.4	2.2	10. Arm Symptoms	1.8	5.6
Top 10 reasons	85.2		Top 10 reasons	83.6	

* Broad Categories of Primary Reason for Visit Codes correspond to ICD chapters

** Wellness was not originally part of the NAMCS Reason for Visit Classification. Most of these visits are for relaxation.

Most visits were for chronic problems, either problems that were ongoing (41% in Connecticut and 32% in Washington) or for flare-ups of chronic problems (12% in Connecticut and 15% in Washington). About a quarter to a third of all visits were for non-illness care (32% in Connecticut and 27% in Washington) and the remainder of visits were for acute problems (15% in Connecticut and 17% in Washington).

### Interaction with other healthcare providers and insurance

Most massage visits resulted from self-referrals (64% or 75%) but 4% in Connecticut and 11% in Washington resulted from referrals by medical or osteopathic physicians (virtually all for musculoskeletal symptoms). Although massage therapists discussed the care of the patient with another provider in 22% of visits in Connecticut and 30% in Washington, that provider was a medical or osteopathic physician less than one-third of the time. The most frequent consultations were with chiropractors. Massage therapists indicated that medical or osteopathic physicians were treating their patients for the same condition for 24% (Connecticut) or 32% (Washington) of visits. Massage therapists noted that they had discussed their patients' care with the physicians of 29% (Connecticut) or 49% (Washington) of their physician-referred patients compared with only 12 – 14% of their other physician-managed patients. Two percent of visits in both states ended with a referral to a medical or osteopathic physician.

Insurance covered only 8% of visits in Connecticut and 26% of visits in Washington, and almost all the remainder were paid for by the patient.

### Care during visits to massage therapists

Massage therapists performed assessments in about two-thirds to three-quarters of the visits (Table 3). The most common methods were tissue assessment via palpation, range of motion, and postural assessment. Multiple assessments were used in 38% (Connecticut) or 48% (Washington) of visits.

**Table 3:** Diagnostic assessments performed by massage therapists licensed in Connecticut (1999) and Washington (1998)

	Connecticut	Washington
	
	(N = 965 visits)	(N = 1040 visits)
*Diagnostic Assessment*	*Percent Using*	

At least one	67.2	74.0
Applied Kinesiology	2.0	5.8
Postural Assessment	19.8	30.7
Range of Motion	34.9	46.0
Tissue Assessment	56.3	60.8
Other (e.g., acupressure point assessment)	7.1	2.7

Virtually all visits included a massage that emphasized at least two techniques (Table 4). The most commonly emphasized techniques were Swedish massage, deep tissue, and trigger point/pressure point techniques. Massage therapists in both Connecticut and Washington emphasized five other techniques in between 14% and 25% of visits: energy work, hot/cold therapy, movement re-education, craniosacral, and reflexology. Massage therapists in Connecticut were more likely to emphasize Oriental bodywork (i.e., meridian based techniques such as shiatsu) while those in Washington were more likely to emphasize neuromuscular therapy. Definitions of some of the most commonly emphasized techniques are provided in Additional File 2.

**Table 4:** Massage techniques emphasized during visits to massage therapists licensed in Connecticut (1999) and Washington (1998)

	Connecticut	Washington
	
	(N = 965 visits)	(N = 1040 visits)
*Techniques Emphasized*	*Percent Using*	

Any	99.4	99.9
Craniosacral	15.3	15.1
Deep Tissue	62.8	65.3
Emotional Bodywork	5.7	4.0
Energy Work	24.9	17.2
Guided Imagery	5.3	4.7
Hot/Cold Therapy	19.9	24.2
Manual Lymph Drainage	3.8	6.3
Movement Re-education	19.2	24.2
Neuromuscular Therapy	5.8	20.5
Oriental Bodywork	16.6	8.6
Pregnancy Massage	1.4	0.7
Reflexology	15.0	15.4
Somatherapy	1.2	5.0
Swedish Techniques	80.6	76.8
Trager	6.7	14.1
Trigger Point/Pressure Point	51.5	45.6
Other (e.g., Esalen, Thai)	7.1	4.2
Two or more techniques	86.7	92.5

More than 80% of visits included self-care recommendations (Table 5), with 50% (Connecticut) or 64% (Washington) of visits including multiple recommendations. Increasing water intake, movement (especially active movement), body awareness, and breathwork were the most common recommendations. Visits lasted a median of 60 minutes.

**Table 5:** Self-care recommendations given by massage therapists licensed in Connecticut (1999) and Washington (1998)

	Connecticut	Washington
	
	(N = 965 visits)	(N = 1040 visits)
*Self-Care Recommendations*	*Percent Using*	

Any	81.1	84.6
Body Awareness	37.2	37.7
Breathwork	28.4	25.2
Hot/Cold Therapy	29.0	33.2
Movement – any	39.2	44.6
*Movement – active*	*26.6*	*35.1*
*Movement – passive*	*17.3*	*13.5*
*Movement – resisted*	*7.2*	*7.8*
Visualization	8.3	8.7
Water Intake, Increase	48.4	56.1
Other (e.g., self-massage, relaxation	5.6	3.4

## Discussion

To our knowledge, this is the first study that describes the demographic and training characteristics of US massage therapists and uses systematically collected visit data to describe their treatment patterns. Strengths of the study are the collection of data from licensed massage therapists practicing in geographically separated parts of the country where CAM use is relatively common, random sampling of providers from state licensing lists, relatively high response rates, and large sample sizes. The main limitation is that we collected data from only two states, which may not be representative of massage practice in other states.

However, licensure requirements in Connecticut and Washington are similar to those in most other states with licensure requirements. As of December, 2004, 33 states and the District of Columbia had passed legislation regulating massage practice. Of those, 21 require exactly 500 hours of training for licensure and 12 require between 570 and 1000 hours [8]. Licensure in both Connecticut and Washington requires 500 hours of training plus a passing score on the national certification exam administered by the National Certification Board for Therapeutic Massage and Bodywork (NCBTMB). The latter is required for licensure in 24 states and is an option for licensure in another 5 states. In some states, including Massachusetts and California, massage regulations vary within the state (i.e., between townships, cities or counties). By contrast, the two provinces in Canada with regulatory requirements mandate that massage therapists receive 2500 hours (Ontario) or 3300 hours (British Columbia) of training.

### Characteristics of the massage therapists

Our study describes an eclectic group of health professionals. Most massage therapists have taken continuing education training that includes both Western-oriented treatment techniques (e.g., neuromuscular therapy, myofascial release), and non-Western oriented treatment techniques (e.g., Reiki, meridian-based massage). Our finding that most massage therapists are white females with a median age around 40 is consistent with the findings of the only other published study of the characteristics of massage therapists, which surveyed 82 massage practices in the Boston area [9]. However, that study reported that the median length of practice was 7 years (compared to our 4 to 5 years), that providers received a median of 1000 hours of clinical training (compared to our 600 hours), and that practitioners saw a median of 20 patients per week (compared to our 10 to 15 visits per week). The other study used the telephone book in a single urban area to recruit massage therapists whereas we used state – wide licensing lists. Their restriction to an urban area, their recruitment methods and their lower response rate may have biased their sample toward busier practitioners.

### Why patients visit massage therapists and evidence for efficacy

The majority of visits to massage therapists focused on musculoskeletal conditions, possibly reflecting the extensive use of massage by physical therapists for rehabilitation during the first half of the 20^th ^century [10]. These are conditions for which Western medical care is often of limited value, which may explain why back and neck pain are the most common reasons why patients seek CAM care in general [2]. While massage as a relaxation technique has received abundant attention in the popular culture, we found that less than one-third of all visits to licensed massage therapists focused on non-illness care.

CAM is also commonly used for self-defined anxiety and depression [2,11]. Among such a group of respondents to a national survey, 5% and 2% of respondents said that they used massage therapy to treat these conditions, respectively [11]. Since massage therapists do not make diagnoses, no information is available on whether patients' visiting for anxiety and depression in our study actually had these disorders diagnosed by physicians.

We could find no other published studies presenting data on patients' reasons for visits to massage therapists from a large population-based sample of visits, so we do not know how comparable these results are. A survey of a representative sample of US adults reported that massage therapy was one of the most common CAM therapies used for back problems, neck problems and fatigue [2]. While fatigue was not a commonly listed reason for visiting massage therapists in our study, some patients who received wellness care or care for anxiety or depression could conceivably have had fatigue as a symptom.

The use of massage for treating medical conditions has grown substantially since 1990 [2]. Although massage is one of the most popular forms of CAM care and has been found to have intriguing physiological effects (reviewed by Field [12]), few studies with moderate to large sample sizes have been conducted to evaluate its clinical effectiveness, even for most musculoskeletal conditions, conditions for which massage is frequently sought and for which conventional medicine has few good treatments. Three recent studies, including two that were well designed and had reasonable sample sizes, evaluated therapeutic massage as a treatment for subacute or chronic back pain and all three found positive results [13]. In addition, several studies of acupressure for back pain have also found positive results [14,15]. A recent Cochrane review of massage for back pain [16] concluded that "massage might be beneficial for patients with subacute and chronic non-specific back pain, especially when combined with exercises and education. More studies are needed to confirm these conclusions". While even fewer studies of massage have been conducted for other musculoskeletal pain conditions, there are small studies suggesting that massage may have benefits for patients with fibromyalgia [17], shoulder pain [18] and diffuse chronic pain [19], while Irnich [20] did not find massage effective for neck pain. Most of those studies lacked follow-up after the treatments had stopped, but Hasson found that the benefits of massage did not persist three months after the last treatment.

A recent meta-analysis of randomized trials of massage for various conditions found that massage had its greatest short-term benefits in reducing trait anxiety and depression, but no studies have evaluated these effects after the end of the treatment period [21]. A systematic review of massage for symptom relief in cancer patients found preliminary evidence that massage had short term benefits on psychological well-being and possibly anxiety [22], but called for additional studies to confirm and extend these findings.

The modest evidence base for massage therapy's clinically important effects provides physicians with little information for advising patients about its effectiveness for conditions other than subacute or chronic back pain. However, given the safety profile and preliminary evidence of effectiveness for back pain, physicians should feel comfortable recommending massage for selected patients with musculoskeletal conditions and, possibly, for mild stress-related anxiety.

### Care during visits to massage therapists

Massage therapists in Washington were more likely than those in Connecticut to use postural assessment and range of motion as assessments tools. Such differences likely reflect differences in training. In general, these differences in assessment were not associated with differences in the massage techniques emphasized by practitioners. Swedish, deep tissue, and trigger (pressure) point were by far the most popular techniques in both states. In their survey of massage therapists in Boston, Lee and Kemper [9] found similar results: 90% of practitioners reported using Swedish techniques and more than half reported using trigger point massage, sports massage, myofascial release, and aromatherapy.

A substantial minority of visits included techniques with a non-Western origin, such as some forms of energy work (e.g., Reiki) and meridian-based massage. In addition, this study as well as a previous study [23], found that massage therapists often emphasize self-care (e.g., drinking more water, movement, body awareness). Recommendations often include increasing the patients' awareness of how they are using their bodies coupled with exercises designed to enhance movement and posture, based on the assumption that many musculoskeletal conditions result from poor use of the body. While these recommendations have not been scientifically validated, they are likely to be safe and may enhance the patient's sense of well-being.

### Safety of massage

In a review of the safety of massage therapy, Ernst [24] found 16 case reports and 4 case series in the biomedical literature over a 6 year period describing adverse effects associated with various forms of massage. However, only 3 reports (including 7 cases) described adverse effects that were probably attributable to treatments by massage therapists practicing Western forms of massage. These included the displacement of a ureteral stent, a hepatic hematoma after deep tissue massage [25] and the deterioration in hearing among patients who received neck massage. Ernst found three additional reports of adverse events associated with shiatsu, the most serious of which was retinal artery embolism with partial loss of vision after application of shiatsu to the upper neck. Although the rate of adverse effects over this period of time is unknown, in the US alone an estimated 113 million visits were made to massage therapists in 1997 [2], suggesting that serious adverse experiences due to massage are extremely rare.

Despite these scattered reports of adverse experiences, common forms of massage (e.g., Swedish, deep tissue, and neuromuscular) are considered very low risk, especially when massage is tailored appropriately to the individual (e.g., possible pressure or anatomic site restrictions), as massage therapists are commonly trained to do [10]. While it is still generally assumed that patients with deep vein thrombosis should not receive massage to the lower extremities, many previous contraindications, such as proscribing massage to patients with metastatic cancer, are no longer considered warranted. Massage therapists are trained not to massage anatomic sites containing localized conditions such as skin injuries or burns.

### Communication between massage therapists and physicians

Massage therapy is an increasingly popular form of care used by patients who are often also being treated by a physician for the same condition. Nevertheless, we found that massage therapists and physicians rarely communicated with each other. Possible barriers to communication include our observation that most patients who see both a physician and a massage therapist for a particular condition were not referred to massage by the physician. Furthermore many massage therapists are not trained in charting language familiar to physicians, nor are they permitted to make "diagnoses". In addition, referring patients to massage therapists has not been part of the training of physicians. Finally, we suspect that most massage therapists, who are typically part-time solo practitioners, lack office staff and record systems to assist with administrative tasks, including routine (and written) communication with other care providers.

We believe that patients may benefit from increased communication between their physicians and massage therapists. Physicians can foster improved communication by asking patients about the care they are receiving from a massage therapist and learning about the treatment plan. Some patients will want to try massage therapy only after consultation with their physician. In these circumstances, physicians can use the framework recommended by Eisenberg [26] to guide patients through the process of selecting a well-trained, therapeutically-oriented massage therapist, jointly negotiating the treatment plan, and monitoring the effects of the treatment over time.

## Conclusion

While substantial barriers to the full integration of massage therapy into the healthcare system remain (e.g., variability between states in licensure and practice regulations, lack of widespread insurance reimbursement, lack of solid studies on efficacy for many frequently-treated conditions, ambivalence on the part of massage therapists as to the advisability of mainstreaming)[27], the information provided in this report should be informative to physicians and other healthcare providers interested in advising their patients about massage therapy.

## Competing Interests

The author(s) declare that they have no competing interests.

## Authors' contributions

KJS participated in the design of the overall project and the data analyses and drafted this manuscript. DCC was the PI on one of the grants funding the study, designed and directed the data collection and analysis of the overall project. JK helped design the data collection instruments. JE participated in the design of the overall project, directed the data collection, quality control, and participated in the analyses for this paper. AH directed the data collection for Connecticut. RD participated in the design of the overall project and data collection procedures and helped to obtain funding. DME was the PI on one of the grants funding the study and participated in the design of the overall project and data collection procedures. All authors read and approved the manuscript.

## Pre-publication history

The pre-publication history for this paper can be accessed here:



## Supplementary Material

Additional File 1 Massage Care Survey. The visit form used for each of the massage therapy visits Click here for file

Additional File 2 Glossary of Selected Massage Techniques. Definitions of selected massage techniques Click here for file
