# Supplementary material for: A survey of training and practice patterns of massage therapists in two US states
Source: BMC Complement Altern Med. 2005 Jun 14;5:13. doi: 10.1186/1472-6882-5-13 (PMC1182347; doi:10.1186/1472-6882-5-13)
Supplement: Additional File 1 — Massage Care Survey. The visit form used for each of the massage therapy visits [file 1472-6882-5-13-S1.doc]

| **Assurance of Confidentiality: All information which would permit identification of an individual, a practice, or an establishment will be held confidential, will be used only by persons engaged in and for the purpose of the survey and will not be disclosed or released to other persons or users for any other purpose.** | | | | | | **MASSAGE CARE SURVEY** PATIENT ENCOUNTER FORM | | | | | | | **ID#: ____ ____ ____ ____/___ ___**  **Provider Patient** | | |
| --- | --- | --- | --- | --- | --- | --- | --- | --- | --- | --- | --- | --- | --- | --- | --- |
| **1. DATE OF VISIT**  _____ / _____ **/ _98** Month Day Year | **2. PATIENT’S BIRTH**  **MONTH & YEAR**  ________ ________ MONTH YEAR | | **3. PATIENT’S SEX** 1q Male 2q Female**î**  **3a. Is patient pregnant?**  1q Yes 2q No3q Unknown | | | | | **4. PATIENT’S RACE**  1q White  2q Black  3q Asian/Pacific Islander  4q American Indian/Eskimo/Aleut | | | **5. PATIENT’S ETHNICITY**  1q Hispanic origin  2q Not Hispanic | | | | **6. DOES PATIENT SMOKE CIGARETTES**?  1q Yes  2q No  3q Unknown |
| **7. PLACE OF VISIT** **Check one** 1q Office outside home  2q Office in home  3q Training clinic  4q Patient’s home  5q Other: Specify______________ ________________ | | 8. PATIENT REFERRED BY *Check one*  1q Self / another patient / friend  2q Acupuncturist  3q Chiropractor  4q Massage therapist  5q Midwife  6q Naturopathic physician  7q Neurologist  8q Other medical physician  9q Osteopathic physician  10q Physical or occupational therapist  11q Other: *Specify* ________________ | | | **9. IF REFERRED BY A PHYSICIAN,**  **WHAT WAS THE PRIMARY DIAGNOSIS FOR WHICH** THE PATIENT WAS REFERRED? 1q Patient not referred by a physician  2q Unknown diagnosis  3q Diagnosis: ____________________________________  9a. ICD-9 CODE OF PRIMARY DIAGNOSIS __ __ __ . __ __ | | | | | **10. PATIENT’S COMPLAINT(S), SYMPTOM(S), OR OTHER**  **REASON(S) FOR THIS VISIT èè *Use patient’s own words***  **Item 10a**. Most important: ___  ___  **b.** Other: ___  ___  **c.** Other: ___  ___ | | | | | |
| 11. HAVE YOU SEEN THIS PATIENT BEFORE  FOR ANY REASON?  1q Yes 2q No *(Skip to #12)*  ê  **11a. For the reason listed in Item 10a?**  1q Yes 2q No *(Skip to #12)*  ê  **11b. Was this an initial or follow-up visit?**  1q Initial visit  2q Follow-up visit | | | | 12. Is this patient receiving care from a medical or osteopathic physician for the reason listed in Item 10a?  1q Yes  2q No  3q Unknown | | | **13. HAVE YOU DISCUSSED THE CARE OF THIS PATIENT WITH**  **ANY OTHER HEALTH PROFESSIONALS WHO ARE PROVIDING CARE FOR THIS PATIENT?**  1q Yes 2q No *(Skip to #14)* 13a. Which type(s) of provider(s)? *Check all that apply* 1q Acupuncturist 6q Naturopathic physician  2q Chiropractor 7q Osteopathic physician  3q Other massage therapist 8q Physical or occupational therapist  4q Neurologist 9q Other: *Specify* __________________  5q Other medical physician | | | | | **14. MAJOR REASON FOR THIS VISIT** Check one 1q Acute problem  2q Chronic problem, routine (on-going)  3q Chronic problem, flare-up  4q Pre- or post-surgery/injury follow-up  5q Non-illness care or wellness care | | | |
| 15. TEST(S) PERFORMED DURING THIS  VISIT *Check all that apply*  1q None  2q Applied kinesiology  3q Postural assessment  4q Range of motion (ROM)  5q Tissue assessment  6q Other: *Specify*_______________________  ______________________________________  **______________________________________** | | | **16. TECHNIQUE(S) EMPHASIZED DURING THIS VISIT** **(GENERAL CATEGORIES)**  1q None 10q Neuromuscular therapy  2q Cranio-Sacral 11q Oriental bodywork (e.g., Shiatsu)  3q Deep tissue (e.g., myofascial release, friction) 12q Pregnancy massage  4q Emotional bodywork (e.g., Rosen) 13q Reflexology  5q Energetic work (e.g., Reiki, therapeutic touch) 14q Somatherapy (e.g., Rolfing®, Hellerwork, Hanna Somatics)  6q Guided imagery 15q Swedish techniques  7q Hot/cold therapy 16q Trager®  8q Manual lymphatic drainage 17q Trigger point/pressure point  9q Movement education/re-education (e.g., MET, 18q Other: *Specify ___________________________________*  Feldenkrais®, Passive ROM, Alexander) | | | | | | | | | **17. SELF-CARE RECOMMENDED DURING THIS VISIT *Check all that apply***  1q None  2q Body awareness  3q Breathwork  4q Hot/cold therapy  5q Movement/exercise: active  6q Movement/exercise: passive  7q Movement/exercise: resisted  8q Visualization  9q Water intake, increase  10q Other: *Specify: __________________* | | | |
| **18. What is your assessment of the areas you treated, related to the patient’s most important reason for visit in Item 10a?**  _____ ________ _____  _____ ________ _____  _____ ________ _____  _____ ________ _____ | | | **19. VISIT DISPOSITION *Check all that apply***  1q No follow-up planned 10q Referred to osteopathic physician  2q Return if needed 11q Referred to physical/occupational  3q Return at specified time therapist  4q Referred to acupuncturist 12q Other:Specify_______________  5q Referred to chiropractor __________________________  6q Referred to other massage therapist  7q Referred to neurologist  8q Referred to other medical physician  9q Referred to naturopathic physician | | | | | | **20. PRIMARY EXPECTED SOURCE OF**  **PAYMENT FOR THIS VISIT *Check on****e*  1q Private insurance  2q Worker’s Compensation (L&I)  3q Medicare  4q Medicaid  5q Personal Injury Protection (PIP)  6q Self-pay  7q No charge  8q Unknown  9q Other *Specify*_____________________ | | | | | **21. Was any of the care you provided at this visit outside the scope of your massage license, but covered by a license you have in another health care profession?**  1q Yes 2q No   1. VISIT DURATION   ___________  Minutes | |

This study is a joint effort of researchers at the Group Health Cooperative of Puget Sound, the University of Washington, the Harvard Center for Alternative Medicine Research and the Centers for Disease Control and Prevention. 4.30.98
