# Supplementary material for: A survey of training and practice patterns of massage therapists in two US states
Source: BMC Complement Altern Med. 2005 Jun 14;5:13. doi: 10.1186/1472-6882-5-13 (PMC1182347; doi:10.1186/1472-6882-5-13)
Supplement: Additional File 2 — Glossary of Selected Massage Techniques. Definitions of selected massage techniques [file 1472-6882-5-13-S2.doc]

**Glossary of Selected Massage Techniques**

**CranioSacral Therapy** (CST), is a gentle, hands-on method of evaluating and enhancing the “function” of the craniosacral system, which consists of the membranes and cerebrospinal fluid surrounding the brain and spinal cord. Using a soft touch that is about the weight of a nickel, practitioners claim to release “restrictions” in this system to improve the function of the central nervous system.

**Deep tissue massage:** involves deeper manipulation of the muscle or surrounding fascia [28]. It may include strokes from Swedish massage with the application of greater pressure and/or greater anatomic specificity, as well as other strokes [1].

**Energy techniques:** techniques that attempt to balance or otherwise adjust the “flow of energy” in the body through either very light touch or holding the hands just above the skin [1]. Some examples of energy techniques are Reiki, Polarity, and Therapeutic Touch.

**Meridian-based massage:** a variety of massage techniques, which were originally developed in East Asia and focus on treating presumed imbalances in the body's energy or “chi”. These meridian-based therapies include acupressure, Amma, and Tuina. Shiatsu (“finger pressure”), an adaptation of acupressure, is the dominant form of Japanese massage.

**Movement re-education:** use of movement to enhance body awareness and movement for the patient [28]. Some styles of movement re-education emphasize active exercises (e.g, Alexander technique) to teach healthier ways of moving. Others emphasize tablework in which the practitioner induces, assists or resists movement for a patient (e.g., “Proprioceptive Neuromuscular Facilitation”, “positional release”, passive and active assisted or resisted exercise and stretch). A third group of movement therapies offers a combination of tablework and exercises (e.g., Trager®, Feldenkrais®).

**Swedish massage:** the most widely taught and practiced style of relaxation massage in the US. It employs five types of strokes: effleurage (gliding), petrissage (kneading and lifting), friction (moving the tissue layers underneath the skin), vibration, and percussion [1, 28].
